# Supplementary material for: Borneo coral reefs subject to high sediment loads show evidence of resilience to various environmental stressors
Source: PeerJ. 2019 Aug 14;7:e7382. doi: 10.7717/peerj.7382 (PMC6698134; doi:10.7717/peerj.7382)
Supplement: Supplemental Information 10 [file peerj-07-7382-s010.docx]

**Supplementary Methods**

Coral samples were removed from the -80 refrigerator and kept on ice for 2 hours to thaw. The protocol for extracting tissue was adapted from Ben-Haim et al. (2003) with some modifications. Samples were carefully rinsed with filtered sea water (FSW) to remove any unrelated material (e.g. sediments, shells etc.) and dried with tissue paper before dry weighing. Samples were kept in their individual plastic bags and tissue was removed from the skeleton through water picking with FSW using a water-gun with pressurized water. Samples were dried and reweighed to estimate the wet tissue weight. Total volume of the used FSW (approx. 100-500 ml) accumulated in each bag was recorded and 4 aliquots of 40 ml each (1 for symbiont analysis, 1 for chlorophyll measurements and 2 as a control) were sampled into 50 ml plastic centrifuge tubes. The aliquots were centrifuged at 9,500 rpm for 40 minutes at 4oC. The supernatant was discarded, and the algal pellets were stored at -20^o^C for further analysis. Difficulties were encountered in obtaining fixed pellets, since some samples contained highly dissolvable pellets. Samples were thus centrifuged for 60 minutes. Another issue was the high mucus production of some coral samples as a stress response to the temperature change. The slimy mucus product trapped the tissue pellet and could not be removed.

**Symbiont density and condition**

The tissue pellet was resuspended in 2 ml of FSW and homogenised in a tissue grinder that had been placed in an ice water bath to ensure separation of the zooxanthellae cells from the tissue. For cell preservation, 2 ml of alcohol (70%) was added to the cell suspension and was kept in 4 degrees until further processing. Prior to microscopy, cell suspensions were mixed by a vortex to dissolve the mucus product that would aggregate cells into large clumps and disable the counting. The number of zooxanthellae cells were counted using the Olympus BX51 microscope and an integrated digital camera Olympus DP70. Counting was done with a with a 20x or 40x magnification and photographs were taken with a 100x magnification on a Neubauer haemocytometer. The last consists of two chambers each containing a grid with a dimension of 5x5 mm and subdivided into 9 squares of 1 mm each. For the counting, the central square was used which was split into 25 squares with a dimension of 0.2 x 0.2 mm each (figure 6a). Per coral sample, 5 replicate counts were carried out by counting zooxanthellae cells across all 25 squares at each chamber. A droplet of approximately 0.5 ml of cell suspension was injected into the haemocytometer and the image was adjusted on a grey scale filter. Cells within the squares and adjacent to the outer walls were counted. Total number of cells was calculated using formula (1) where Naverage is the average count between the 5 replicates and Vtissue is the volume used by the waterpicking to remove the tissue. The density was calculated using formula (2). We multiplied by 10,000 since each square has a volume of 0.1 μl and the whole grid has a volume of 1 ml.

Ntotal = Naverage x Vtissue x 10,000 (1)

Dzoox = Ntotal / SA (2)

To estimate zooxanthellae health for each coral samples, the counting was carried out as for the zooxanthellae density with difference being that every symbiont cell was categorized in a degradation scale from 1 to 5 as follows: 1 = cell is healthy, 2= initiation of degradation (cell-wall starts to disintegrate), 3= in degradation process (cell wall has been disrupted), 4= degraded and dead cell (content has been dissolved), 5= totally degraded (some contents to

be seen in form of bubbles; Supp fig 1).

**Chlorophyll a content**

Measuring pigments in the symbiont cells is a good early warning indicator of nitrification in waters (Marubini et al., 1999; Fabricius et al., 2005b-b) and essential to be measured together with sediment dynamics to understand the factors causing health decline. The second tissue pellet was resuspended in 10 ml of FSW and homogenized with a tissue grinder on an ice bath in the dark. The resulting suspension was centrifuged at 9,500 rpm for 30 minutes in 4 degrees. The supernatant was discarded, and the pellet resuspended in 6 ml of 90% acetone and left in dark for 20-24 hours. The next day, the algal suspension was centrifuged at 8,000 rpm, at 4 degrees for 12 minutes. Chlorophyll a absorbance was measured with an SHIMAZU, UVmini-1240 automated spectrophotometer at the following wavelengths: λ664, λ647, λ630 and λ691. Glass cuvettes were used to avoid fogging by the acetone solution. Chlorophyll a content was calculated using equation (3) derived from Jeffrey et al. (1975)

Chlorophyll a (μg/ml) = 11.85 E664 – 1.54E647 – 0.08E630 (3)

**Surface area estimations**

For assessing the surface area of the coral samples, the popular wax coating method was used (Strickland, 2010). According to Naumann et al. (2009), this technique has an intermediate accuracy but performs well in estimating surface areas for branching forms. Additionally, it is time and cost efficient. Coral samples were dipped for 1 second into melted paraffin wax that was kept at 70oC and were weighed to the nearest 0.01 g. This was repeated and the weight of the second wax (W2) coating was calculated by subtracting it from the first weight (W1). Calibration objects (cubed coral samples of different sizes) were used as a reference to estimate the surface area (SA) of the unknown samples. A photograph was taken of each face of the calibration objects and images were imported in the software CPCe for surface area calculations. These data were used to produce a reference graph of known surface area against weight (r2= 0.96). The equation of the trendline (y =ax + b) of this graph was then used to calculate the SA of unknown coral fragments as in formula (4).

SA = [a x (W2-W1)] + b (4)
